# Draft genomic sequence of *Enterobacter hormaechei* ML02 isolated from mangrove-associated rhizosphere soil in Bangladesh

**DOI:** 10.1128/mra.00024-26

**Published:** 2026-04-27

**Authors:** Sabbir Ahmed, Md. Anisul Kabir, Md Ashikuzzaman Antor, Abu Reza, Naeema Salatia Hoque, Fateha Nahum Tahia, Md. Minhajul Abedin, Muslima Rahman Shrabony, Surma Perveen, Mohammad Abu Hena Mostofa Jamal

**Affiliations:** 1 Department of Biotechnology, Faculty of Engineering Sciences, German University Bangladesh 421870 , Gazipur, Bangladesh; 2 Laboratory of Medical and Environmental Biotechnology, Islamic University 247297, Kushtia, Bangladesh; 3 Department of Environmental Science and Geography, Faculty of Sciences, Islamic University 247297, Kushtia, Bangladesh; 4 Department of Biotechnology and Genetic Engineering, Faculty of Biological Sciences, Islamic University 247297, Kushtia, Bangladesh; 5 Department of Genetic Engineering and Biotechnology, Faculty of Health and Life Sciences, Daffodil International University 833161 , Dhaka, Bangladesh; 6 Department of Pharmacy, Faculty of Biological Sciences, Islamic University 247297, Kushtia, Bangladesh; Montana State University, Bozeman, Montana, USA

**Keywords:** genome sequence, *Enterobacter hormaechei* ML02, rhizosphere soil, mangrove

## Abstract

This study reports the draft genome sequence of *Enterobacter hormaechei* ML02, isolated from mangrove-associated rhizosphere soil in Bangladesh. The assembled genome is 4,874,798 base pairs in length, distributed across 80 contigs, with a GC content of 55.5%, and encodes 4,565 predicted protein-coding genes.

## ANNOUNCEMENT

*Enterobacter hormaechei* is a gram-negative member of the *Enterobacter cloacae* complex, taxonomically defined in 1989 (1). Like other *Enterobacter* spp., *E. hormaechei* occupies soil, aquatic, plant-associated, and animal gastrointestinal environments, reflecting broad ecological adaptability (2–4). Genomic analysis and whole-genome sequencing (WGS) of rhizobacteria identify plant growth-promoting, stress tolerance genes, and biosynthetic gene clusters (5). This study presents the draft genome assembly of *Enterobacter hormaechei* ML02.

*Enterobacter hormaechei* ML02 was isolated in 2020 from a soil sample from the rhizosphere of Mangrove Flora in Bangladesh (22° 30′ 00″ N, 89° 39′ 36″ E). One gram of soil was suspended in 9 mL sterile 0.85% (weight per volume) NaCl, serially diluted to 10⁻⁶, and 100-µL aliquots were spread onto Tryptic Soy Agar (TSA) (HiMedia, India). Plates were incubated at 28 °C for 72 h, and isolates were purified by streaking on fresh TSA. Genomic DNA from a single *Enterobacter hormaechei* ML02 colony, cultured in Tryptic Soy Broth for 24 h, was extracted using the GF-1 Soil Sample DNA Extraction Kit (6). After cell lysis, the lysate was centrifuged at 14,000 rpm for 5 min to remove debris, and genomic DNA was purified from the supernatant using the GF-1 PCRClean up Kit (7). Genomic DNA quantity and purity were measured using the NanoDrop spectrophotometer and Qubit 4.0 Fluorometer, with Qubit working solution prepared at 1:200 dilution (8). Polymerase chain reaction (PCR) amplification was performed using gene-specific primers on a Bio-Rad T100 Thermal cycler (Bio-Rad, Hercules, USA) (9). Then, 300 ng of genomic DNA was used for library preparation with the Illumina DNA Library Preparation (Prep) Microbial (M) Tagmentation kit (96 samples) (10). Whole-genome sequencing of *Enterobacter hormaechei* ML02 was conducted at the Genomic Center, icddr,b, Dhaka, Bangladesh, using the Illumina NextSeq 550 platform with a NextSeq 500/550 High-Output Kit v2.5 (300 cycles), generating 2 × 150 bp paired-end reads.

Raw sequencing reads were demultiplexed using bcl2fastq v2.20.0 (Illumina) (11). Raw paired-end reads were quality-trimmed using Sickle v1.33 (minimum length 20) (12) and assembled with SPAdes v4.0 (13). Pilon v1.24 polished the assembly (14). The assembled genome was submitted to PATRIC under BV-BRC v3.55.17 for detailed genomic analysis (15). The genome was annotated using the National Center for Biotechnology Information (NCBI) Prokaryotic Genome Annotation Pipeline (PGAP) v.6.9 (16, 17). The identity of the bacterium was confirmed by Sanger sequencing of the 16S rRNA gene (18). BLASTn comparison of *Enterobacter hormaechei* ML02 16S rRNA sequences with NCBI confirmed species identity via high similarity (19). 16S rRNA and whole-genome-based taxonomic analysis of *Enterobacter hormaechei* ML02 was conducted using the Type (Strain) Genome Server (TYGS) (https://tygs.dsmz.de) (20). Both 16S rRNA and whole-genome phylogenies confirmed the classification of strain ML02 as *Enterobacter hormaechei*, with the closest 16S rRNA gene sequence accession number CP140425.1. Default parameters were used for all software tools in this study.

The draft genome of *Enterobacter hormaechei* ML02 spans 4,874,798 base pairs in 80 contigs, with 98.38× coverage and 55.5% GC content. A total of 4,686 genes, including 4,565 protein-coding sequences, were predicted. Key genomic features are summarized in Table 1.

**TABLE 1:** Genomic features of *Enterobacter hormaechei* ML02

Features	Value
Description of the source	
Location	Sundarbans, Bangladesh
Time	2022
Type	Rhizosphere Soil
Sequencing summary	
Coverage	98.38×
Genome length	4,874,798 base pairs
Number of raw reads	1,919,339 base pairs
GC content (%)	55.5
Number of replicons	34
Total bases	566 megabases
Size	224.9
Assembly report	
Tool used	SPAdes v. 4.0
Contigs	80
Genome size	4.9 megabases
Contig L50	4
Contig N50	466.4 kilobases
Annotation report	
Tool used	PGAP
Genes (total)	4,686
CDS (total)	4,620
Genes (coding)	4,565
CDSs (with protein)	4,565
Genes (RNA)	66
rRNA	1, 3, and 5 (5S, 16S, and 23S)
ncRNAs	5
tRNA	52
CDSs (without protein)	55

## Data Availability

The Whole-Genome Shotgun sequence for this study is available in GenBank under accession number JBJXVC000000000.1. The raw sequencing reads are deposited in the Sequence Read Archive database under accession number SRR31360097. Both data sets are linked to BioSample number SAMN44754504 and BioProject number PRJNA1186382. The assembled genome is readily available under the accession number GCF_046244455.1/. The 16S rRNA gene is PZ052326.
